# The Contribution of Missense Mutations in Core and Rim Residues of Protein–Protein Interfaces to Human Disease

**DOI:** 10.1016/j.jmb.2015.07.004

**Published:** 2015-08-28

**Authors:** Alessia David, Michael J.E. Sternberg

**Affiliations:** Centre for Integrative Systems Biology and Bioinformatics, Department of Life Sciences, Imperial College London, SW7 2AZ London, United Kingdom

**Keywords:** ASMT, acetyl serotonin *O*-methyltransferase, PDB, Protein Data Bank, SPT, serine pyruvate aminotransferase, PLCE1, 1-phosphatidylinositol 4,5-bisphosphate phosphodiesterase epsilon-1, PPI, protein–protein interaction, SAV, single amino acid variation, SSE, secondary structure elements, protein–protein interaction, core and rim interface, human disease, SAVs, nsSNPs

## Abstract

Missense mutations at protein–protein interaction sites, called interfaces, are important contributors to human disease. Interfaces are non-uniform surface areas characterized by two main regions, “core” and “rim”, which differ in terms of evolutionary conservation and physicochemical properties. Moreover, within interfaces, only a small subset of residues (“hot spots”) is crucial for the binding free energy of the protein–protein complex.

We performed a large-scale structural analysis of human single amino acid variations (SAVs) and demonstrated that disease-causing mutations are preferentially located within the interface core, as opposed to the rim (*p* < 0.01). In contrast, the interface rim is significantly enriched in polymorphisms, similar to the remaining non-interacting surface. Energetic hot spots tend to be enriched in disease-causing mutations compared to non-hot spots (*p* = 0.05), regardless of their occurrence in core or rim residues. For individual amino acids, the frequency of substitution into a polymorphism or disease-causing mutation differed to other amino acids and was related to its structural location, as was the type of physicochemical change introduced by the SAV.

In conclusion, this study demonstrated the different distribution and properties of disease-causing SAVs and polymorphisms within different structural regions and in relation to the energetic contribution of amino acid in protein–protein interfaces, thus highlighting the importance of a structural system biology approach for predicting the effect of SAVs.

## Highlights

• Protein–protein interactions are fundamental in all biological processes.
• The distribution of deleterious and non-SAVs within protein interfaces is unknown.
• The distribution of deleterious SAVs differs within different interface structural regions.
• The distribution of SAVs differs in relation to interface residues energetic contribution.
• Structural analysis of protein complexes enhances the understanding of deleterious SAVs.

## Graphical abstract

**Figure:**
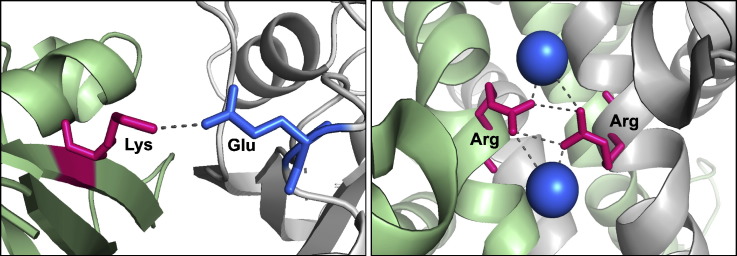

## Introduction

With more than a million individual-specific DNA variations identified in the human genome, deriving insights from the large quantity of data generated by genome-wide association studies, individual whole genome sequencing and, in the near future, the 100,000 genomes project are a major challenge.

Protein–protein interactions (PPIs) are central in all biological processes and understanding how PPIs contribute to human disease is essential. As proteins do not function in isolation but interact in complex biological networks, single amino acid variations (SAVs) occurring at protein interfaces can profoundly disrupt not just a single protein but an entire biological pathway. Protein–protein recognition and interaction occurs in specific areas of the protein surface, known as protein interfaces. We previously showed that protein interfaces are enriched in disease-causing missense mutations compared to other protein surface regions [1], a finding that was confirmed by others [2,3]. The important contribution of amino acid changes occurring at protein interfaces to human disease has recently been corroborated by studies showing that mutations causing cancer occur at protein interaction sites more often than they occur on the rest of the protein surface [4]. Mutations can disrupt a protein interface by modifying its physicochemical, structural and energetic characteristics [3,5–7]. Teng *et al.* recently studied a large set of protein complexes and showed that disease-causing mutations occurring at protein interfaces tend to alter the free binding energy of the complex, compared to harmless SAVs [8].

A protein interface is a complex, non-uniform area located on the protein surface. PPI occurs through interactions between residues on two opposite interfaces [9,10]. Although a protein interface can occupy a large area, only a small subset of its residues plays a crucial role in the binding free energy of the complex. These few key residues are known as energetic “hot spots” [11–13] and are typically identified *in vitro* and *in silico* as those residues that cause a ≥ 2 kcal/mol reduction in binding free energy when mutated to alanine [12].

The characteristics of hot spots have been extensively studied. It has been shown that they are enriched in arginine, tryptophan and tyrosine and they overlap to a large extent with interface “core” residues [14]. Interface core residues become solvent inaccessible upon PPI, as opposed to interface “rim” residues that remain partially solvent accessible [15,16]. The interface core and rim differ not only in their energetic contribution to the complex stability but also in terms of their physicochemical and evolutionary characteristics. Interface core residues tend to be evolutionarily more conserved and their side chains often display less mobility upon binding, compared to rim residues [17,18]. A more complex partition of the interface proposed by Levy identifies a third region called support, which amino acid composition resembles that of the protein buried (interior) region [19].

Despite the clear distinctive functional importance of core and rim residues, as well as that of energetic hot spots *versus* less energetically important residues, the individual contribution of core and hot spot residues to human disease remains largely unexplored. One could expect to see rare disease-causing mutations and common polymorphisms differentially distributed between interface residues. Moreover, disease-causing mutations are expected to have a greater impact on protein structure, function and protein complex thermodynamics when occurring in core residues or in energetic hot spots.

In this study, we performed a systematic analysis of the distribution and properties of naturally occurring polymorphisms and disease-causing SAVs within interface core and rim residues and compared these findings to those obtained from SAVs localized on the remaining protein structure. Moreover, we analyzed the effect of polymorphisms and disease-causing SAVs on the complex binding free energy.

## Results

### SAVs are differentially distributed according to their location within the interface

**Fig. 1:**
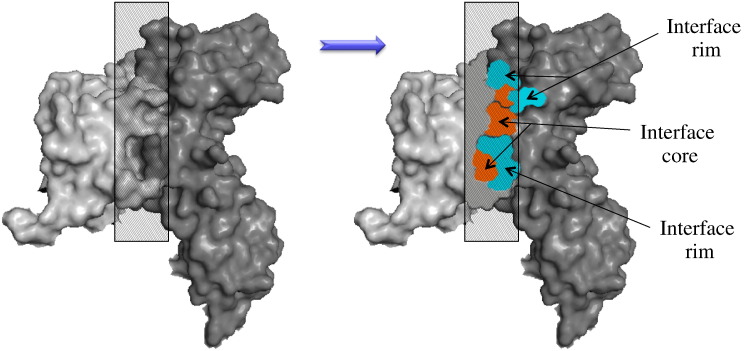
Schematic diagram of core and rim interface regions. Highlighted is a cross-sectional view of a protein–protein interface. Interacting proteins are presented in light and dark gray, respectively. The interface core is presented in orange and the rim is presented in blue.

A total of 3282 disease-causing SAVs and 1699 polymorphisms occurring in 705 proteins were analyzed. A total of 62.6% of all SAVs (disease and polymorphisms) occurred in solvent-accessible residues, which comprise interacting (interface) and non-interacting (surface) amino acids. Within disease-causing SAVs, 54.5% (1788 SAVs) occurred in solvent-accessible residues, of which 466 were located on the protein interface (215 in the core and 251 in the rim) and 1322 were located on the remaining protein surface. Disease-causing SAVs were 35% more likely to occur at interfaces compared to non-interacting surfaces (OR 1.35, 95% CI 1.22–1.51, *p* < 0.0001) but were 39% less likely to occur in interface than in buried residues (OR 0.61, 95% CI 0.55–0.67, *p* < 0.0001), in keeping with earlier work by David *et al.* [1]. To provide a structural relationship between protein interface anatomy and human genetic variations, we divided the interface in core and rim (Fig. 1). Disease-causing SAVs were 49% more likely to occur in the interface core rather than the rim and were 72% more likely to occur in the interface core than in the non-interacting protein surface (Table 1), thus clearly demonstrating a different contribution of core and rim regions to human disease. Moreover, disease-causing SAVs were not preferentially located at the interface rim rather than the remaining non-interacting surface.

When polymorphism location was analyzed, 78.7% of polymorphisms resided within surface-accessible residues (241 in interface residues and 1096 in surface non-interface residues). Polymorphisms were less likely to be located in the interface core compared to the rim (*p* < 0.003) or the rest of the non-interacting surface (*p* < 0.0002). No difference was detected in the frequency of polymorphisms between the interface rim and the rest of the non-interacting surface (*p* = 0.65).

The results of our previous study [1] suggested that the interacting and non-interacting surface had similar enrichment in polymorphisms, which appeared to be in contrast to the interface enrichment in disease-causing SAVs. Dividing the interface in core and rim demonstrated that the enrichment in polymorphisms is in the rim and not in the core. The interface rim and the non-interacting surface are characterized by lower evolutionary conservation [17] and more side-chain flexibility [18] compared to the interface core and the protein buried regions, thus explaining why the interface rim and the non-interacting surface have a greater ability to accommodate amino acid changes.

### *In silico* prediction of disease-causing SAVs and polymorphisms varies across different protein regions

**Fig. 2:**
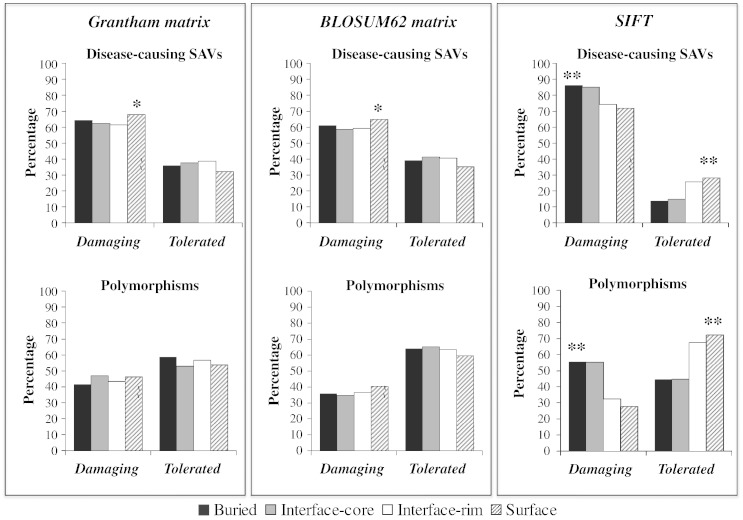
Distribution of the percentages of BLOSUM62, Grantham and SIFT scores for 3282 disease-causing SAVs and 1699 polymorphisms according to their location in different protein regions. Substitutions were characterized as “damaging” according to the following parameters: Grantham score > 60, BLOSUM62 score < 0 and SIFT score ≤ 0.10. *, Bonferroni-corrected *p* value of < 0.05 ; **, Bonferroni-corrected *p* value of < 0.01.

Residue conservation and the physicochemical characteristics of disease-causing SAVs and polymorphisms occurring in different protein regions were analyzed using BLOSUM62 and Grantham matrices and SIFT (Sorting Tolerant From Intolerant). BLOSUM62 and Grantham matrices score substitutions are based on multiple sequence alignment and physicochemical changes from the wild type, respectively. Both methods showed that the majority of disease-causing SAV scores were compatible with a deleterious effect. Moreover, when the effect of disease-causing SAV was analyzed, both matrices showed that the number of SAV identified as deleterious was significantly greater for disease-causing SAV located on the non-interacting surface compared to SAVs located in any other protein region (Bonferroni-corrected *p* value of < 0.05, χ^2^ test; Fig. 2). Surface residues are less evolutionarily conserved and not under the constraint of tight interior packing and restricted conformational space, as in the case of buried residues, and can, therefore, accommodate larger physicochemical changes compared to buried and interface core residues. In contrast, buried and interface residues have highly specialized roles and subtle changes in size or physicochemical properties may be sufficient to alter protein stability and function. Thus, a more substantial change in the properties of a residue at the non-interacting surface will generally be required to cause an alteration of protein biological activities. When BLOSUM62 and Grantham matrices were used to analyze polymorphisms, the majority of SAVs had scores suggestive of a “tolerant” nature. No significant difference in score distribution was observed across the four protein regions when BLOSUM62 and Grantham scores indicative of radical changes were used (a score less than or equal to − 2 for BLOSUM62 and greater than 100 for Grantham).

When disease-causing SAVs were analyzed using SIFT, the number of SAVs identified as deleterious was significantly greater for disease-causing SAVs located in the buried region compared to disease-causing SAVs in other regions, whereas the number of deleterious SAV identified as tolerant was significantly greater for disease-causing SAVs located in non-interacting surface compared to disease-causing SAVs in the other regions (Bonferroni-corrected *p* < 0.01, χ^2^ test; Fig. 2). The distribution of SIFT scores for polymorphisms was similar, with a significantly greater number of disease-causing SAVs predicted as damaging when located in buried residues compared to surface and interface rim residues (Bonferroni-corrected *p* < 0.01, χ^2^ test; Fig. 2). SIFT, similarly to other heavily used prediction programs, scores substitution using position-specific information obtained from the alignment of homologous proteins, as functionally and structurally important amino acids are under evolutionary constraint, especially within a single protein family. The less evolutionarily conserved nature of residues in interface rim and non-interacting surface compared to residues in buried and interface core regions may explain why deleterious substitutions in these regions were less likely to be correctly predicted by programs such as SIFT, which rely heavily on sequence conservation among homologues.

### Amino acid mutability varies across different protein regions

Available prediction methods have been shown to have different sensitivities in predicting the deleterious effect of substitutions at individual amino acid levels [20]. Arginine and glycine are the most important contributors to disease [21–24]. Nevertheless, arginine and glycine are not among the top three amino acids for which widely used prediction servers exhibit the highest prediction sensitivity [20]. We determined whether the structural localization of amino acid could add information in terms of predicting the effect of SAVs.

**Fig. 3:**
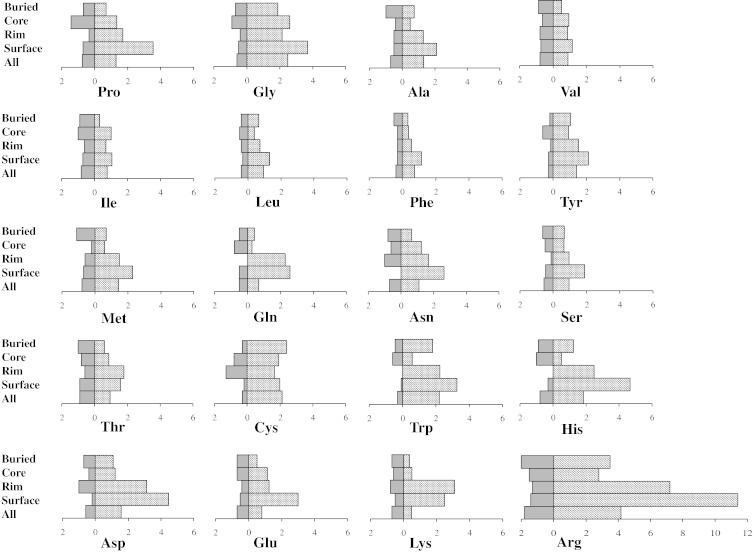
Amino acid susceptibility to disease-causing SAVs or polymorphisms according to structural location. The percentages of disease-causing SAVs (striped boxes) and polymorphisms (uniform dark-gray boxes) harbored by each amino acid across the entire protein (“All”) and within the four structural regions of the protein, that is, buried (Buried), interface core (Core), interface rim (Rim) and non-interacting surface (Surface), are shown.

Disease-causing SAV and polymorphism frequencies for each of the 20 amino acids were analyzed according to their location in the whole protein sequence and in the four structural regions (Fig. 3). Although individual amino acid susceptibility to harbor a deleterious SAV or a polymorphism varied across different protein regions, Arg, Gly and Trp harbored more disease-causing SAVs than polymorphisms, whereas the opposite was true for Val and Ile, Glu, Lys and Thr (Supplementary Tables 1 and 2).

In our dataset, Arg and Gly contributed 29% of all disease-causing mutations and 20% of all polymorphisms, which is in accordance with previous finding [25]. Mutations in codons encoding Arg result in substitution of Arg with amino acids with very different chemical characteristics. A recent study by Petukh *et al.* looking at the most frequent disease-causing mutations showed that a substantial proportion involved a change from Arg to Cys, Pro or Trp [21]. Among the most frequent harmful amino acid substitutions were also changes from Gly to Arg, Asp, Glu and Val [25]. Glycine is the smallest of the 20 amino acids and its substitution with any other residue is likely to have a major impact on protein structure. Trp has an important, quite specialized structural and functional role, such as stacking interactions, and its replacement is poorly tolerated. Moreover, because of its large size, substitutions of Trp are likely to generate large cavities in protein structure, which can compromise protein stability, as well as affect PPIs.

Since arginine is an important contributor to human disease [23], we explored the effect of arginine substitutions in the four different protein regions. As expected, arginine was the most mutable amino acid (4.1% of all arginines in the dataset harbored a disease-causing SAV and 1.8% harbored a polymorphism) and the main contributor to disease-causing SAVs (529 disease-causing SAVs were arginine substitutions, 16.1% of all disease-causing SAVs). Nevertheless, when structural regions were analyzed separately, arginine substitutions were the main contributors to disease in all but the buried region. Glycine substitutions were the main contributors to disease in the buried region, which can be explained as this region is under spatial constrain and substitution of glycine is likely to result in steric clashes. This is particularly true when considering that the most harmful glycine changes include substitutions with large amino acids, such as Arg, Asp and Glu [25].

When we examined the percentage of substitutions relative to each individual amino acid across the different regions (Supplementary Table 1), a disease-causing SAV was harbored by 11.1% of buried and by 7.2% of interface core arginine residues. These frequencies were well above the median across all amino acids for each region (buried amino acids, median of 2.38 and range of 11.1–1.0; interface core amino acids, median of 1.7 and range of 7.2–0.5) and vastly higher than what was observed for arginine harboring disease-causing SAVs located in interface rim and non-interacting surface residues (2.8% and 3.5%, respectively). Moreover, the disease-causing *versus* polymorphism ratio was 8.1, 11.7, 1.9 and 1.76 for arginine substitutions occurring in buried, interface core, interface rim and non-interacting surface, respectively, thus reflecting the important structural and functional role of arginine in interface core and buried regions, compared to the remaining protein structure. Arginine structural role in proteins includes formation of salt bridges and hydrogen bonds, alteration of which has been shown to occur more in harmful than harmless amino acid changes [3,25].

### SAV characteristics vary across different protein regions

**Fig. 4:**
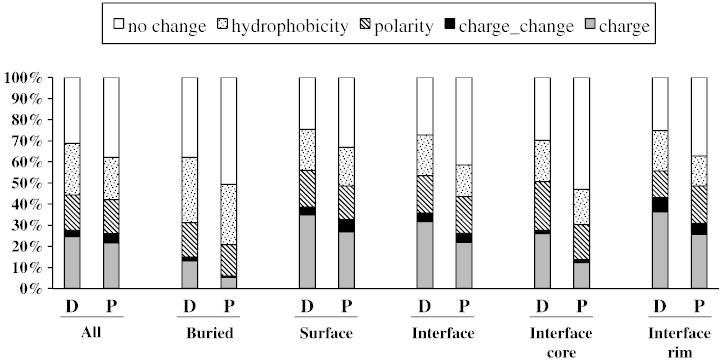
Distribution of the physicochemical changes introduced by SAVs. Changes were evaluated in terms of hydrophobicity, charge or polarity loss and charge change across the different protein regions and overall (All). Data were analyzed for disease-causing SAVs and polymorphisms. D, disease-causing SAVs; P, polymorphisms. Surface, non-interacting surface.

We next evaluated the physicochemical changes introduced by SAVs in terms of hydrophobicity, loss of charge or polarity and charge change (Fig. 4). Disease-causing SAVs resulted in a major change in 69.0% of all cases, which, unsurprisingly, was a significantly higher proportion (*p* < 0.01) compared to polymorphisms (major change observed in 62.1% of cases). This was in agreement with a recent study, which showed that the less harmful amino acid changes are those that introduce minimal changes in the physicochemical characteristics of the residue [25]. Moreover, we found that the distribution of physicochemical changes was significantly different between disease-causing SAVs and polymorphisms, overall (*p* < 0.01) and according to specific protein region (*p* < 0.01). Overall, significantly more disease-causing SAVs resulted in a loss of hydrophobicity (*p* = 0.045) and significantly fewer disease-causing SAVs resulted in a change in charge, compared to polymorphisms (*p* < 0.01).

Data were subsequently analyzed in relation to the location of SAVs on the protein. A significant difference in the distribution of change types across the four protein regions was present (*p* < 0.01), reflecting the different structural and functional role of amino acids in individual regions. SAVs causing hydrophobicity loss were more likely to occur in buried residues (462 out of 807) than in other regions of the protein (*p* < 0.01), which reflects the importance of hydrophobicity in driving correct protein folding. Loss of hydrophobicity in buried amino acids is a well-known cause of protein misfolding and early degradation [26]. SAVs causing loss of charge or a change in charge (from positive to negative or vice versa) were more likely to occur in the non-interacting surface and interface rim, whereas SAVs causing loss of polarity were more frequent in the interface core compared to the remaining regions. Electrostatic interactions are the driving force of PPIs and introduction of mutations altering the side-chain polarity and charge can profoundly affect complex stability.

We did not examine the distribution of SAVs in relation to their location in secondary structure elements (SSE). Nevertheless, a recent study that correlated the degree of harmfulness of naturally occurring amino changes to their location according to SSE showed that neutral and deleterious SAVs were equally distributed among SSE. The authors concluded that SSE location was not useful for the prediction of the deleterious effect of SAV, whereas the type of amino change was more informative [25]. This is in agreement with our results, which underlined the importance of analyzing the location (e.g., interface *versus* non-interacting surface) and type of change introduced by SAVs (e.g., changes in the amino acid charge or in the ability to form salt bridges compared to wild type) when predicting the deleterious effect of mutations.

### Interface hot spots tend to be enriched in disease-causing SAVs

The energetic contribution to protein complex stability is not uniform across the interface but is generally provided by a small subset of energetically important “hot spot” residues [14,27]. In order to address whether hot spots are enriched in disease-causing SAVs, we first predicted the contribution of interface residues to the binding free energy of protein–protein complexes. This was achieved by performing alanine scanning mutagenesis on the interface residues of 2298 PPIs in our dataset. The energy distribution across interface residues, classified as core and rim, is presented in Supplementary Fig. 1. Interface core residues contributed significantly more than rim residues to the binding free energy of the complex, regardless of the threshold used. In particular, 42.5% of interface core residues *versus* 23.2% of interface rim residues had ΔΔ*G*_WT_ ≥ 1 kcal/mol (*p* < 0.001, χ^2^ test) and 20.3% *versus* 8.9% had ΔΔ*G*_WT_ ≥ 2 kcal/mol (hot spot residues, *p* < 0.001, χ^2^ test).

Subsequently, we examined the energetic change introduced by mutations. Energy data were predicted for 366 disease-causing SAVs and 187 polymorphisms located at interface core and rim residues. Disease-causing SAVs occurred preferentially in interface residues with ΔΔ*G*_WT_ ≥ 1 kcal/mol rather than < 1 kcal/mol (*p* < 0.002) (Table 2). Within different energetic classes, SAVs occurred more often in core rather than rim residues (*p* < 0.01). Moreover, a trend was observed (*p* = 0.0508) for disease-causing SAVs to occur in hot spots (ΔΔ*G*_WT_ ≥ 2 kcal/mol), regardless of their localization in core or rim residues (Table 2). Although our results did not reach statistical significance, possibly because of the small number of observations, interface hot spots appear to be important contributors to human disease. When polymorphisms were analyzed, no preferential location in interface residues with ΔΔ*G*_WT_ ≥ 1 kcal/mol rather than < 1 kcal/mol was found.

Next, we examined the energetic changes introduced by SAVs. Disease-causing SAVs destabilizing the protein–protein complex (ΔΔ*G*_MUT_ ≥ 2 kcal/mol) were preferentially located in hot spot residues (ΔΔ*G*_WT_ ≥ 2 kcal/mol, *p* < 0.001), whereas disease-causing SAVs causing a modest or no change in binding free energy (ΔΔ*G*_MUT_ < 1 kcal/mol, *p* < 0.001) were preferentially located in energetically less important residues, and this was independent from residue location (interface core *versus* rim, *p* = 0.07). Similar results were obtained for polymorphisms. As the presence of structures generated by homology modeling in our dataset may have biased the accuracy of energy prediction, the abovementioned analyses were repeated using data available from experimentally solved human structures only, confirming our results (Supplementary Tables 3 and 4).

In our dataset, 17 polymorphisms (9 in interface core and 8 in the rim) were predicted to cause a major change in binding free energy (ΔΔ*G*_MUT_ ≥ 2 kcal/mol). Since this was an unexpected finding, the phenotypes associated with these 17 polymorphisms were analyzed in detail and results are presented in Supplementary Table 5. Twelve of seventeen polymorphisms were located in energetic hot spots. Minor allele frequency data were available for seven polymorphisms and were indicative of a rare polymorphism (minor allele frequency of < 0.05) in three cases. Phenotypic data were available for 3 of 17 polymorphisms. Two SAVs were reported to be associated with a reduction in protein function and one SAV had no effect at protein level. The paucity of experimental data does not allow us to exclude that the remaining 14 polymorphisms, which are predicted to have an impact on protein complex stability, may have a subtle effect at phenotype level, alone or in combination with other SAVs, such as the case of p.Pro11Leu (dbSNP: rs34116584) in the serine pyruvate aminotransferase (SPT; UniProt: P21549). This common polymorphism is a Pro-to-Leu substitution at the interface rim of the SPT homodimer. It creates a new mitochondrial binding site, which is hidden and, thus, inefficient in the normally tightly folded homodimer. Nevertheless, in approximately 5% of cases, the SPT protein can translocate to the mitochondria [28], thus suggesting that p.Pro11Leu can have a subtle destabilizing effect on the homodimer.

We next examined whether the energy contribution of the wild-type residue (ΔΔ*G*_WT_) may help predict the nature of the SAV. We found that the ΔΔ*G*_WT_ was a poor predictor of the nature of SAVs (polymorphism or disease-causing) and significantly underperformed compared to SIFT and two prediction servers Polyphen2 [29] and SuSPect [30], as assessed by ROC analysis (data not shown).

### Examples of the biological impact of SAVs occurring at the interface rim

In our study, 257 (7.7%) disease-causing mutations and 175 (10.3%) polymorphisms occurred in the interface rim. The following are examples of the biological impact of SAVs occurring in this structural location, which is historically considered less important than the interface core.

**Fig. 5:**
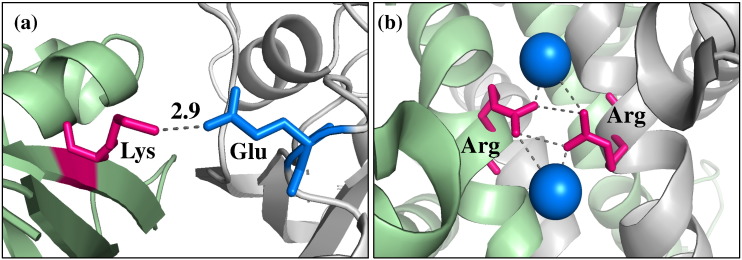
Interface rim SAVs associated with disease. (a) Mutation p.Glu63Lys. The wild-type glutamic acid 63 located at the interface rim of the GTPase HRAS protein is presented in blue and the interacting lysine on protein PLCE1 is presented in magenta (PDB ID: 2c5l). (b) Polymorphism p.Arg115Trp (dbSNP: rs201053197). The water-mediated interaction between wild-type arginines 115 at the ASMT homodimer interface is shown. Interacting arginines are presented in magenta and water molecules are presented as blue spheres (PDB ID: 4a6d). The two chains of the interacting proteins are presented in green and gray in both cases. Distances between atoms of two interacting residues are calculated in angstroms (Å).

The deleterious mutation p.Glu63Lys has been identified in the GTPase HRas protein (UniProt: P01112) of patients with Costello syndrome (MIM: 218040) [31]. The latter is characterized by several severe congenital abnormalities, which range from mental retardation to cardiac malformations. GTPases of the RAS family are important regulators of the 1-phosphatidylinositol 4,5-bisphosphate phosphodiesterase epsilon-1 (PLCE1; UniProt: Q9P212). Activation of the latter triggers an intracellular cascade, ultimately leading to cell growth and differentiation. Glutamic acid at position 63 (Glu63) in the GTPase HRas protein is highly conserved, is not predicted to be an energetic hot spot and is located at the interface rim between proteins GTPase HRas and PLCE1 (Fig. 5a). Glu-to-Lys substitution is predicted to be damaging by SAV prediction servers (Polyphen2 score = 0.983, SuSPect score = 98, SIFT score = 0.00). Our structural analysis reveals that substitution of the negatively charged Glu with the positively charged Lys is predicted to abolish the formation of a salt bridge with Lys located on protein PLCE1, thus potentially destabilizing the interaction between these two proteins.

Polymorphism p.Arg115Trp (dbSNP: rs201053197) occurs in acetyl serotonin *O*-methyltransferase (ASMT; UniProt: P46597), an enzyme involved in the synthesis of melatonin. Amino acid substitutions in this enzyme are a known risk factor for the development of autism [32]. Low melatonin levels are reported in autistic patients and are associated with a reduction in ASMT activity in patients with ASMT variations [32]. p.Arg115Trp is a rare polymorphism identified in autistic patients [33]. Arg115 is located at the rim of the large ASMT homodimerization interface (Fig. 5b). Arg115 is not predicted to be an energetic hot spot and its substitution to Trp is predicted benign (Polyphen2 score = 0.235, SuSPect score = 14, SIFT score = 0.18). Arg115 is not conserved across homologous proteins. Nevertheless, it is difficult to assess the contribution of evolutionary conservation to disease prediction when dealing with disorders such as autism, for which no animal model exists. We performed a structural analysis, which showed that Arg-to-Trp substitution can abolish a water-mediated polar interaction between Arg115 on the two ASMT chains, as well as generate a steric clash, thus potentially resulting in a less favorable interaction and impaired protein function. This highlights that a detailed structural analysis can have a central role in the identification of SAVs that are likely to be disease-causing variants.

## Discussion

Extensive analysis of interface residues in a large dataset of protein–protein complexes allowed us to demonstrate that disease-causing SAVs occur significantly more often in the interface core rather than in the rim, whereas the opposite is true for polymorphisms. The interface core is considered to be instrumental in establishing the affinity and stability of PPI [17]. Because of their highly specialized functional and structural role, interface core residues, similarly to residues in the hydrophobic buried protein region, tend to be evolutionarily conserved among homologous proteins, and amino acid substitutions in these regions are less likely to be tolerated compared to those occurring in the interface rim and non-interacting surface.

Studies have shown that disease-causing amino acid changes are found mostly in buried and interface core residues [1,25]. Although our study shows that the interface rim and non-interacting surface regions are not enriched in deleterious SAVs, their contribution to disease is not to be underestimated. In our dataset, more than one half of disease-causing SAVs occurred in these two regions, in agreement with previous reports [34]. This underlines the importance of correctly predicting deleterious mutations occurring in these protein areas. The interface rim and non-interacting surface are generally less conserved from an evolutionary point of view compared to the remainder of the protein structure and can generally accommodate larger substitutions in terms of physicochemical changes, as shown by our results using BLOSUM62 and Grantham matrices. This can explain the lower sensitivity of SIFT and other widely used prediction servers in predicting the deleterious effect of substitutions occurring in solvent-exposed residues, compared to buried residues [20,35]. The ability to computationally discriminate between neutral and deleterious variants can greatly aid in prioritizing candidate SAVs for additional studies and can contribute in improving SAV prediction programs. Structural data can provide important information and help establish the phenotypic impact of amino acid substitutions occurring in solvent-accessible areas, such as the non-interacting surface and interface rim.

In our study, arginine substitutions were 16.2% of all disease-causing mutations, which is similar to what has been previously reported (19%) [23]. Arginine is the most mutable amino acid [23] and its substitutions account for a large proportion of the most harmful mutations [25]. Our results suggest that its substitutions are likely to be deleterious and this was particularly true for arginine residues located in buried or interface core regions. In these regions, the disease-to-neutral SAV ratio for arginine substitutions was 1:8 and 1:5, respectively, whereas the ratio was less than 2:1 in the non-interacting surface and interface rim. Arginine can form salt bridges and hydrogen bonds, which play the critical role of stabilizing monomeric protein structures, as well as protein–protein complexes [3,36]. Because of this important structural role, arginine substitutions in interface core and buried amino acids are hardly ever tolerated and are likely to have a profound impact on phenotype, as suggested by a recent study, which showed that more than 60% of the most harmful amino acid changes (the majority of which were arginine substitutions) disrupted hydrogen bonds formed by the wild-type amino acids [25].

It has been suggested that protein–protein interfaces should, generally, be energetically optimized to favor complex formation [37] and the negative effect of interface SAVs on the binding energy of protein complexes has been demonstrated for mutations causing diseases, such as glioblastoma [3,4]. A recent analysis of the effect of a large set of experimentally generated amino acid changes obtained from Skempi and ProTherm databases showed that random amino acid changes can destabilize the binding free energy of these engineered proteins. Moreover, the energetic change, although moderate on average, can become major (≥ 2 kcal/mol) for specific amino acid changes [25]. The authors showed that, for randomly placed amino acid changes, the largest energetic change was produced by hydrophobic-to-charged or hydrophobic-to-polar substitutions. Interestingly, our results showed that hydrophobic substitutions are not predominant in protein interfaces. This reinforces the concept that the deleterious effect of an amino acid substitution should be investigated, taking into account the structural context in which the mutation occurs, not just the type of amino acid change introduced.

Energetic hot spots are the major contributors to the binding free energy of protein complexes. When we examined their contribution to human disease, we found that they are enriched in disease-causing substitutions compared to less energetically important interface residues. Nevertheless, our results did not reach the threshold of statistical significance, possibly because of the paucity of data and, thus, need to be confirmed on a larger set of protein interfaces.

Early work on five protein complexes reported in ASEdb suggested that mutations in the interface rim are silent or moderately affect the binding energy of the complex (ΔΔ*G* < 2 kcal/mol), whereas mutations with the most deleterious effect on binding energy (> 2 kcal/mol) occur mainly in interface core residues [38]. A recently published study, which was conducted in parallel to ours, analyzed the effect of the binding and folding energies of a large set of experimentally generated amino acid changes occurring in interface core, rim and support, as well as in the buried (interior) and surface protein regions. The authors showed that the probability of an amino acid substitution to cause a large change in binding free energy (Pp index) more than doubles when the change occurs in a core residue compared to a rim residue [25]. Our results confirm that the interface core provides the majority of hot spots and is enriched in disease-causing SAVs, but they also suggest that minority of rim residues are important contributors to the binding free energy of the complex and substitutions occurring at these positions are not always energetically silent.

One limitation of our study is that we did not assess whether our findings are influenced by the transient or permanent nature of the protein complex, as this would require that proteins were systematically assigned to these two groups, a task that is not trivial. A second limitation of our study could be the use of a single program to predict the energetic effect of SAVs. Although we could have verified our results by implementing other energy prediction programs, a recent study showed that combining results obtained using different methods does not significantly improve the results obtained from individual methods [39].

The use of structural data allowed us to perform an in-depth analysis of the mechanisms by which SAVs can affect protein function. Such information, which cannot be derived by current SAV prediction methods, can help to screen for deleterious SAVs, especially in the presence of contradictory results from different prediction programs. Determining the structure–function relationship of PPIs and the impact of SAVs occurring at interfaces is crucial to understand how deleterious mutations and rare polymorphisms can affect biological pathways and can provide a better understanding of the genotype–phenotype relationship.

## Materials and Methods

### Datasets

Human SAVs data were retrieved from UniProt (humsavar.txt, release 23_10) [40]. Each SAV in humsavar is classified as “disease” (SAV with known disease association), “polymorphism” (SAV with no known disease association) and “unclassified” (SAV with too little information to be classified). Unclassified SAVs were not included in the analysis, as their nature remains uncertain.

Human PPI data and protein complexes structural data were retrieved from Interactome3D [41]. Human protein interaction data in this database are derived from experimentally documented human interactions, listed in nine major interaction databases. Additional human PPIs are inferred from experimental data observed in orthologous proteins. Interactome3D provides structural data for human PPIs that are derived from the Protein Data Bank (PDB) [42]. In the absence of an experimental structure for the human complex, structures are derived from homologous complexes [41]. To ensure that all models for complexes were reliable, we required that the sequence identity between the homologue in PDB and UniProt was ≥ 30%. In addition, SAVs analysis was only performed on proteins for which at least 80% of the UniProt sequence was covered by the PDB.

### Calculation of protein interfaces, relative solvent-accessible area and binding free energy

Residues were assigned to interface when the distance between at least one atom on two different interacting proteins was within 5 Å. The percentage relative solvent-accessible area of each amino acid was calculated by dividing its total surface area with that in the extended conformation (ф = Ψ = 180°) of the Gly–X–Gly tripeptide. Residues were defined as “buried” or “solvent-accessible” residues according to relative solvent-accessible area, using a cutoff of 7% [43]. Interface residues were segregated in core and rim according to their solvent accessibility in the bound state. Residues were defined as core when they became buried upon PPI and were defined as rim when they remained partially exposed [15] (illustrated in Fig. 1).

For each interface residue, the contribution to the binding free energy of the protein complex was calculated using the FoldX algorithm [44]. Initially, all PDB files were repaired (as recommended by the FoldX protocol) using the RepairPDB built-in function in FoldX, which corrects poor torsion angles and van der Waals clashes, when present. Thereafter, all interface residues in each complex were sequentially mutated to alanine (alanine scanning mutagenesis) and the energetic contribution of each wild-type residue (ΔΔ*G*_WT_) was calculated as the difference between the contribution of alanine and that of the wild-type residue, expressed in kilocalories per mole (kcal/mol). The contribution of each interface residue to the binding free energy of the complex was classified as follows: (1) minor contribution when 0.5 kcal/mol ≤ ΔΔ*G*_WT_ < 1 kcal/mol; (2) moderate contribution (“warm residues”) when 1 kcal/mol ≤ ΔΔ*G*_WT_ < 2 kcal/mol [45]; and (3) major contribution (energetic hot spot residue) when ΔΔ*G*_WT_ ≥ 2 kcal/mol [14]. Calculation of the impact of interface SAVs on the binding free energy of the complex (ΔΔ*G*_MUT_) was performed using FoldX. FoldX has a reported correlation of 0.81 with an SD (FoldX accuracy) of 0.46 kcal/mol between calculated and experimental ΔΔ*G* values when calibrated using the experimentally determined mutational free energy changes of more than 1000 SAVs [44]. FoldX was chosen among other prediction programs because it is easy to use and one of the best prediction programs to evaluate the energetic impact of SAVs [39]. In order to study the effect of SAVs, all repaired PDB structures were mutated using the BuildModel function in FoldX. The algorithm was run several times for each specified mutation using the option “< numberOfRuns>5” to optimize the minimum energy conformation of residues with multiple rotamers. The ΔΔ*G*_MUT_ was calculated as the difference between the binding free energy of the mutant complex and that of the wild type.

### Prediction of the effect of SAVs

Three *in silico* sequence-based methods were applied for predicting the effect of each SAV on protein structure and function:

(1) BLOSUM62 matrix [46]: this method calculates the substitution frequency for all amino acid pairs, based on multiple sequence alignment. A positive score of ≥ 0 is indicative of a conservative substitution, whereas a negative score of less than or equal to − 1 indicated a non-conservative substitution, hence a potentially deleterious SAV. “Radical” changes were those with a score of less than or equal to − 2.
(2) Grantham matrix [47]: this method classifies amino acid changes into classes of increasing physicochemical dissimilarity. In particular, it scores an amino acid substitution based on side-chain atomic composition, polarity and volume. It measures variations in terms of difference in side-chain atomic composition, polarity and volume between two amino acids. Changes were considered “conservative” for scores 0–60 and “non-conservative” when more than 60. Radical changes were considered those with a score of > 100.
(3) SIFT (Sorting Tolerant From Intolerant) [48]: this method predicts the functional impact of a residue change based on position-specific information (position-specific scoring matrices) obtained from the alignment of homologous proteins. Score ranges from 0 to 1. Scores were classified as “intolerant” when ≤ 0.10 and “tolerant” when > 0.10.

### Amino acid classification

Amino acids were divided into the following four groups according to their physicochemical properties: polar (Gln, Asn, His, Ser, Thr, Tyr, Cys, Met and Trp), hydrophobic (Ala, Ile, Leu, Phe, Val, Pro and Gly) and charged (Arg, Lys, Asp and Glu). Residues in the latter group were also divided in positively (Arg and Lys) and negatively charged (Asp and Glu). Although other amino acid classifications could have used (e.g., according to size), this simple classification was used as it allows classifying all amino acids without resulting in amino acid overlap between classes.

### Statistical analysis

All statistical analyses were performed using the statistical packages in R version 3.1.1. The likelihood of a disease-causing or polymorphism SAV residing in a specific region *i* rather that region *j* was expressed in terms of odds for each region (probability of the SAV residing in the region divided by the probability that it does not reside in the region) and comparison between regions made by means of odds ratia: OR*_ij_* = (*x_i_*/(1 − *x_i_*))/(*x_j_*/(1 − *x_j_*)), where *x_i_* is the probability of observing a SAV in region *i*.

The chi-square test was used to compare the observed number of SAVs in each region to the expected if SAVs were distributed according to the number of residues in the different regions. A two-tailed *p* value less than 0.05 was considered indicative of statistical significance. Bonferroni correction was used to correct for multiple comparisons.

### Accession numbers

The following are the accession numbers from four databases: UniProt: P21549, P01112, Q9P212 and P46597; PDB: 2c5l and 4a6d; MIM: 218040; and dbSNP: rs34116584 and rs201053197.

## Figures and Tables

**Table 1:** Odds ratio for disease-causing SAVs and polymorphisms according to protein structure location.

Disease-causing SAVs	Total residues	Observed	Expected	O/E		OR	95% C.I.	p-Value	Adjusted p value
**Buried**	72467	1494	947.05	1.58	**Buried versus Surface**	2.24	2.08 – 2.40	< 0.00001	< 0.00001
**Core**	13486	215	176.24	1.22	**Core versus Buried**	0.77	0.67 – 0.89	0.0004	0.002
**Rim**	23478	251	306.83	0.82	**Core versus Rim**	1.49	1.24 – 1.80	< 0.00001	< 0.00001
**Surface**	141704	1322	1851.88	0.71	**Core versus Surface**	1.72	1.50 – 1.99	< 0.00001	< 0.00001
**Total**	251135	3282			**Rim versus Surface**	1.15	1.00 – 1.31	0.047	0.28
					**Rim versus Buried**	0.51	1.45 – 0.59	< 0.00001	< 0.00001

Polymorphisms	Total residues	Observed	Expected	O/E		OR	95% C.I.	p-Value	Adjusted p value

**Buried**	72467	362	490.26	0.74	**Buried versus Surface**	0.64	0.58 – 0.73	< 0.00001	< 0.00001
**Core**	13486	66	91.24	0.72	**Core versus Buried**	0.98	0.75 – 1.27	0.88	
**Rim**	23478	175	158.84	1.10	**Core versus Rim**	0.65	0.87 – 0.49	0.003	0.018
**Surface**	141704	1096	958.67	1.14	**Core versus Surface**	0.63	0.49 – 0.81	0.0002	0.001
**Total**	251135	1699			**Rim versus Surface**	0.96	0.82 – 1.13	0.65	
					**Rim versus Buried**	1.50	1.25 – 1.79	0.016	0.096

Core, interface core; rim, interface rim; O/E, observed/expected; OR, odds ratio; 95%CI, 95% confidence intervals; p-value, uncorrected p-value; adjusted p-value, Bonferroni adjusted p-values. Surface, non-interacting protein surface.

**Table 2:** Disease-causing SAVs distribution according to energy contribution of interface residues ΔΔ*G*_WT_

	Total residues	Observed	Expected	O/E	*p* Value
ΔΔ*G*_WT_ ≥ 1 kcal/mol	366	171	142.89	1.20	0.002
ΔΔ*G*_WT_ < 1 kcal/mol	29,704	11,569	11,597.11	1.00	
Total	30,070	11,740			

ΔΔ*G*_WT_ ≥ 2 kcal/mol	366	72	58.40	1.23	0.0508
ΔΔ*G*_WT_ < 2 kcal/mol	29,704	4627	4739.60	0.98	
Total	30,070	4699			

Residues with predicted ΔΔ*G*_WT_ ≥ 2 kcal/mol (calculated with alanine scanning mutagenesis, using FoldX) were considered energetic hot spots.
