# Supplementary material for: The Contribution of Missense Mutations in Core and Rim Residues of Protein–Protein Interfaces to Human Disease
Source: J Mol Biol. 2015 Aug 28;427(17):2886–98. doi: 10.1016/j.jmb.2015.07.004 (PMC4548493; doi:10.1016/j.jmb.2015.07.004)
Supplement: Supplementary file 1 — Supplementary material. [file mmc1.docx]

**Supporting information**

**Supplementary Table 1. Contribution of each amino acid to disease-causing SAVs in the whole protein dataset (ALL) and in individual protein regions (Buried, Interface core, Interface rim, Surface).** The absolute number (Abs N) represents the total number of disease-causing SAVs observed for the specific amino acid in the whole protein (ALL) or in a specific protein region, whereas the ‘ % Rel ’ represents the percentage relative to the amino acid frequency in the whole protein (ALL) or in a specific protein region. ‘ % Rel ’ is calculated as the number of disease-causing SAVs for amino acid X in region *100 / total n. of amino acids X in region, where ‘region’ is the whole protein or each of individual protein regions (Buried, Interface core, Interface rim, Surface). E.g. for Arginine Abs N, and % Rel in Buried region are calculated as follows:

Total n. of wild type Arginine residues harbouring disease-causing SAVs = 113

Total n. of arginine residues in Buried regions = 1014

*Abs N.* = 113

*% Rel* = (113*100)/1014 = 11.144

The main contributors in each group are highlighted in bold.

|  | **ALL** | | **Buried** | | **Interface Core** | | **Interface Rim** | | **Surface** | |
| --- | --- | --- | --- | --- | --- | --- | --- | --- | --- | --- |
|  | ***Abs N.*** | ***% Rel*** | ***Abs N.*** | ***% Rel*** | ***Abs N.*** | ***% Rel*** | ***Abs N.*** | ***% Rel*** | ***Abs N.*** | ***% Rel*** |
| **Arg** | ***529*** | ***4.103*** | 113 | ***11.144*** | ***35*** | ***7.157*** | ***62*** | ***2.794*** | ***319*** | ***3.476*** |
| **Gly** | ***424*** | ***2.415*** | ***186*** | 3.647 | ***20*** | 2.139 | ***32*** | ***2.528*** | ***186*** | 1.804 |
| **Leu** | ***238*** | 0.951 | **158** | 1.303 | 13 | 0.737 | 6 | 0.388 | 61 | 0.636 |
| **Ala** | ***237*** | 1.312 | ***158*** | 2.090 | 14 | 1.325 | 5 | 0.466 | 60 | 0.711 |
| **Asp** | ***209*** | 1.554 | 72 | 4.458 | 16 | ***3.119*** | 20 | 1.208 | 101 | 1.045 |
| **Val** | ***156*** | 0.882 | 103 | 1.142 | 10 | 0.883 | 9 | 0.951 | 34 | 0.517 |
| **Pro** | ***152*** | 1.270 | 71 | 3.543 | 10 | 1.706 | 15 | 1.187 | 56 | 0.690 |
| **Ser** | ***150*** | 0.957 | 69 | 1.900 | 7 | 0.950 | 9 | 0.636 | 65 | 0.657 |
| **Glu** | ***133*** | 0.796 | 39 | 3.009 | 6 | 1.250 | 22 | 1.046 | 66 | 0.514 |
| **Thr** | ***120*** | 0.930 | 53 | 1.572 | 14 | 1.802 | 10 | 0.855 | 43 | 0.567 |
| **Tyr** | ***115*** | 1.370 | 53 | 2.096 | 10 | 1.397 | 9 | 0.941 | 43 | 1.025 |
| **His** | ***111*** | 1.788 | 53 | ***4.573*** | 7 | 2.491 | 4 | 0.523 | 47 | 1.174 |
| **Cys** | ***109*** | 2.125 | 53 | 1.993 | 5 | 1.650 | 5 | 1.923 | 46 | ***2.412*** |
| **Asn** | ***108*** | 1.088 | 41 | 2.632 | 7 | 1.687 | 14 | 1.240 | 46 | 0.674 |
| **Ile** | ***104*** | 0.761 | 75 | 1.032 | 7 | 0.714 | 7 | 1.000 | 15 | 0.318 |
| **Met** | ***84*** | 1.439 | 57 | 2.283 | 7 | 1.480 | 3 | 0.580 | 17 | 0.723 |
| **Phe** | ***81*** | 0.738 | 60 | 1.150 | 5 | 0.538 | 3 | 0.393 | 13 | 0.319 |
| **Lys** | ***75*** | 0.488 | 14 | 2.482 | 7 | ***2.692*** | 10 | 0.497 | 44 | 0.351 |
| **Trp** | ***75*** | 2.193 | 39 | 3.258 | 6 | 2.247 | 2 | 0.585 | 28 | 1.735 |
| **Gln** | ***72*** | 0.683 | 27 | 2.488 | 9 | 2.296 | 4 | 0.290 | 32 | 0.416 |

**Supplementary Table 2** **Contribution of each amino acid to polymorphisms in the whole protein dataset (ALL) and in individual protein regions (Buried, Interface core, Interface rim, Surface).** The absolute number (Abs N) represents the total number of polymorphisms observed for the specific amino acid in the whole protein (ALL) or in a specific protein region, whereas the ‘ % Rel ’ represents the percentage relative to the amino acid frequency in the whole protein (ALL) or in a specific protein region. ‘ % Rel ’ is calculated as the number of polymorphisms for amino acid X in region *100 / total n. of amino acids X in region, where ‘region’ is the whole protein or each of individual protein regions (Buried, Interface core, Interface rim, Surface). The main contributors in each group are highlighted in bold.

|  | **ALL** | | **Buried** | | **Interface Core** | | **Interface Rim** | | **Surface** | |
| --- | --- | --- | --- | --- | --- | --- | --- | --- | --- | --- |
|  | ***Abs N.*** | ***% Rel*** | ***Abs N.*** | ***% Rel*** | ***Abs N.*** | ***% Rel*** | ***Abs N.*** | ***% Rel*** | ***Abs N.*** | ***% Rel*** |
| **Arg** | ***231*** | ***0.179*** | 14 | 1.381 | 3 | 0.613 | ***33*** | ***1.487*** | ***181*** | ***1.979*** |
| **Val** | ***137*** | ***0.078*** | ***65*** | ***0.721*** | ***9*** | ***0.794*** | 6 | 0.634 | 57 | 0.867 |
| **Ala** | 130 | 0.072 | 40 | 0.529 | 5 | 0.473 | 4 | 0.373 | 81 | 0.977 |
| **Thr** | 116 | 0.090 | 29 | 0.860 | 5 | 0.644 | 9 | 0.770 | 73 | 0.962 |
| **Gly** | 110 | 0.063 | 26 | 0.510 | 4 | 0.428 | 11 | 0.869 | 69 | 0.684 |
| **Glu** | 109 | 0.065 | 6 | 0.463 | 2 | 0.417 | 15 | 0.713 | ***86*** | ***0.670*** |
| **Ile** | 105 | 0.077 | ***50*** | ***0.688*** | 6 | 0.612 | 7 | 1.000 | 42 | 0.891 |
| **Lys** | 100 | 0.065 | 3 | 0.532 | 2 | 0.769 | 12 | 0.596 | 83 | 0.662 |
| **Leu** | 95 | 0.038 | 39 | 0.322 | ***7*** | ***0.397*** | 8 | 0.518 | 41 | 0.427 |
| **Pro** | 88 | 0.074 | 14 | 0.699 | 2 | 0.341 | ***18*** | ***1.424*** | 54 | 0.666 |
| **Ser** | 88 | 0.056 | 17 | 0.468 | 1 | 0.136 | 7 | 0.495 | 63 | 0.637 |
| **Asp** | 81 | 0.060 | 3 | 0.186 | 5 | 0.975 | 6 | 0.362 | 67 | 0.694 |
| **Asn** | 69 | 0.070 | 0 | 0.000 | 4 | 0.964 | 7 | 0.620 | 58 | 0.850 |
| **Gln** | 52 | 0.049 | 5 | 0.461 | 0 | 0.000 | 11 | 0.798 | 36 | 0.468 |
| **His** | 48 | 0.077 | 3 | 0.259 | 2 | 0.341 | ***18*** | ***1.424*** | 54 | 0.666 |
| **Met** | 48 | 0.082 | 18 | 0.721 | 3 | 0.634 | 1 | 0.193 | 26 | 1.106 |
| **Phe** | 43 | 0.039 | 16 | 0.307 | 3 | 0.323 | 2 | 0.262 | 22 | 0.541 |
| **Tyr** | 22 | 0.026 | 7 | 0.277 | 1 | 0.140 | 6 | 0.628 | 8 | 0.191 |
| **Cys** | 17 | 0.033 | 6 | 0.226 | 4 | 1.320 | 2 | 0.769 | 5 | 0.262 |
| **Trp** | 10 | 0.029 | 1 | 0.084 | 0 | 0.000 | 2 | 0.585 | 7 | 0.434 |

**Supplementary Table 3** **Distribution of** **disease-causing** ***SAVs occurring in energetically neutral (ΔΔG_WT_ <1 kcal/mol) interface residues according to their effect on binding free energy. Data were calculated* from experimentally solved human structures.** ***ΔΔG_WT_*** , predicted wild-type residue contribution to the binding free energy of the complex; ***ΔΔG_MUT_ ,*** predicted change introduced by the SAV on binding free energy of the complex. O/E, Observed -Expected ratio.

**Supplementary Table 4 Distribution of disease-causing** ***SAVs occurring in hot spot (ΔΔG_WT_* ≥ 2Kcal/mol*) interface residues according to their effect on binding free energy. Data were calculated* from experimentally solved human structures.**

***ΔΔG_WT_*** , predicted wild-type residue contribution to the binding free energy of the complex; ***ΔΔG_MUT_ ,*** predicted change introduced by the SAV on binding free energy of the complex. O/E, Observed -Expected ratio.

**Supplementary Table 5:** Characteristics of 17 polymorphisms, which are predicted to cause a major change in binding free energy (ΔΔG_MUT_ ≥2 kcal/mol).

Id, Uniprot Id; MAF, minor allele frequency; Hot spot, a residue is classified as energetic hot spot (Y, yes) if its predicted contribution to the binding free energy (ΔΔG_WT_) calculated by FoldX is ≥ 2 kcal/mol, otherwise not (N).

Phenotype, phenotypic data associated with SAV and reported in Uniprot; §, Reduction of specific activity in vitro, causes mistargeting when associated with R-170; §§, No effect on enzyme activity; §§§, Decreased enzyme activity.

D, deleterious; T, tolerant. n.a., not available.

**Supplementary Figure 1.** Energy (ΔΔG_WT_) distribution across wild-type interface residues, classified as core and rim.
